# Complete Modular Disconnection between AFX2 and VELA Leading to a Type IIIa Endoleak Two Years after Endovascular Aneurysm Repair

**DOI:** 10.3400/avd.cr.25-00129

**Published:** 2026-03-31

**Authors:** Kotaro Mukasa, Yasunori Yakita, Ryosuke Marushima, Musashi Tsuda, Shinichiro Abe, Soichi Asano

**Affiliations:** Department of Cardiovascular Surgery, Chiba Cardiovascular Center, Ichihara, Chiba, Japan

**Keywords:** abdominal aortic aneurysm, endoleak, migration

## Abstract

The AFX endograft (Endologix, Irvine, CA, USA) is known to have a high incidence of late type III endoleaks. We report a 76-year-old male who developed a type IIIa endoleak due to complete modular separation 2 years after endovascular aneurysm repair with an AFX2 main body and a VELA extension. Open surgical graft replacement was performed, and the postoperative course was uneventful. This case demonstrates that in the presence of a large aneurysm diameter or significant neck angulation, even Instructions for Use-compliant and recommended overlap may be insufficient. It suggests the necessity of securing greater overlap and implementing stricter follow-up.

## Introduction

Endovascular aneurysm repair (EVAR) has become an established treatment for abdominal aortic aneurysm (AAA), particularly in elderly and high-risk patients. However, concerns regarding its long-term durability remain a major issue. In particular, the US Food and Drug Administration has issued safety warnings about late-onset type III endoleaks associated with the AFX endograft system (Endologix, Irvine, CA, USA). We report a case of aneurysm sac enlargement due to a type IIIa endoleak that developed 2 years after EVAR with an AFX endograft. This complication progressed rapidly between 1 and 2 years after EVAR and resulted from complete separation between the AFX2 main body and the VELA suprarenal extension graft, ultimately necessitating open surgical graft replacement. Patient data, including imaging and clinical history, were reviewed from our electronic database. The patient provided written consent for publishing the case, including images.

## Case Report

**Fig. 1:**
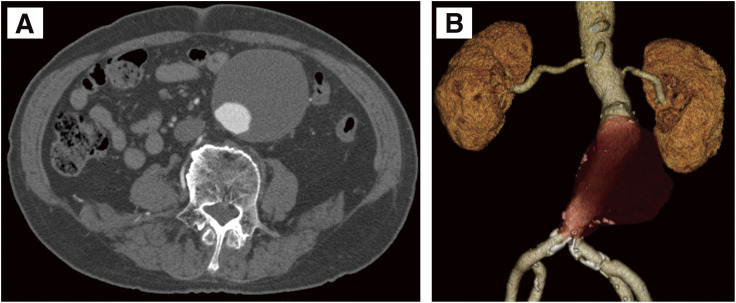
Preoperative contrast-enhanced computed tomography. (**A**) Axial image showing a maximum short-axis diameter of 65 mm. A large amount of mural thrombus is present within the aneurysm sac. (**B**) Three-dimensional reconstruction demonstrating a longitudinal length of 85 mm. The proximal neck measures 37 mm in length with an angulation of 55°, and the aneurysm sac contains extensive mural thrombus.

**Fig. 2:**
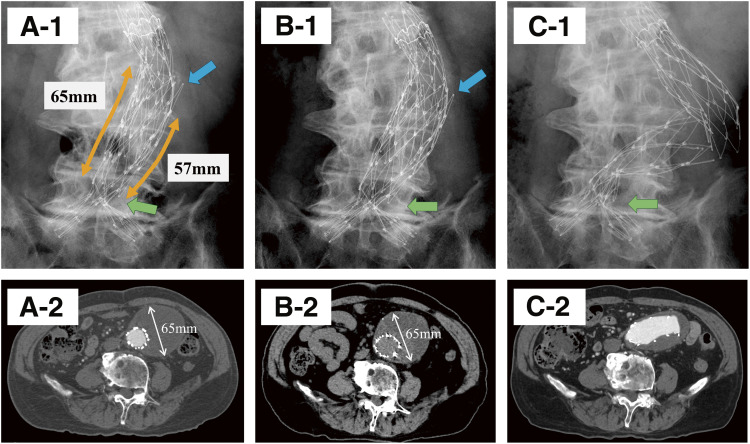
Follow-up imaging. (**A-1**) Plain abdominal radiograph obtained immediately after the procedure, showing a shorter overlap on the greater curvature side of the curved segment. Blue arrow: overlap between the AFX2 main body and the VELA suprarenal extension graft. Green arrow: segment between the fourth segment of the main body and the bifurcation. (**A-2**) Contrast-enhanced computed tomography obtained immediately after the procedure. (**B-1**) Plain abdominal radiograph at 1 year postoperatively, demonstrating a slight reduction in the overlap between the AFX2 main body and the VELA suprarenal extension graft (blue arrow). The distance between the fourth segment of the main body and the bifurcation appears mildly shortened (green arrow). (**B-2**) Plain computed tomography at 1 year postoperatively, showing no change in aneurysm diameter compared with the immediate postoperative imaging. (**C-1**) Plain abdominal radiograph at 2 years postoperatively, demonstrating complete modular disconnection between the AFX2 main body and the VELA suprarenal extension graft. The distance between the fourth segment of the main body and the bifurcation is markedly shortened (green arrow). (**C-2**) Contrast-enhanced computed tomography at 2 years postoperatively.

A 76-year-old male was referred for treatment of an infrarenal AAA identified during screening computed tomography (CT). The aneurysm was fusiform, with a maximum diameter of 65 mm and a longitudinal length of 85 mm (**Fig. 1**). The proximal neck measured 37 mm in length with an angulation of 55°. The distal aorta at the aortic bifurcation measured 18 mm in diameter, which prompted the selection of the AFX endograft. Although the large aneurysm size raised concerns about secure fixation between the AFX2 main body and the VELA extension, it was assumed that the presence of extensive mural thrombus would mitigate the risk of migration. EVAR was performed using a long AFX2 main body (BEA25-90-116) and a suprarenal VELA extension (A28-28-C95-020V). Intraoperative angiography and postoperative CT revealed no evidence of an endoleak. The required overlap based on the manufacturer's recommendation (aneurysm radius +20 mm) was calculated to be 52.5 mm. The overlap was shorter on the outer curvature side than on the inner curvature side. Nevertheless, an overlap length of 57 mm was secured, which was considered sufficient at the time (**Fig. 2**).

**Fig. 3:**
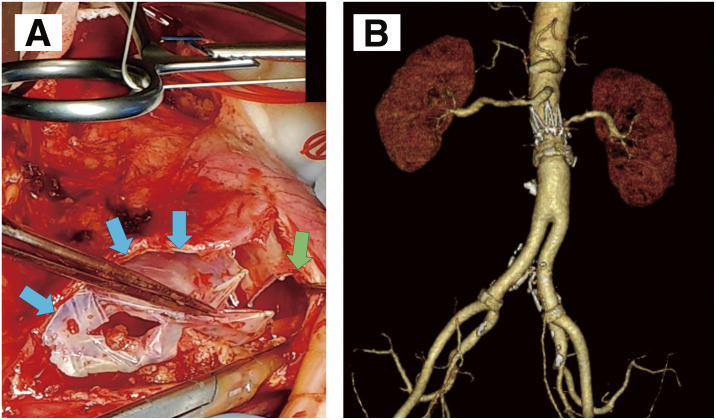
(**A**) Intraoperative photograph after midline laparotomy and opening of the aneurysm sac. Complete disconnection is confirmed at the modular junction between the AFX2 main body and the VELA suprarenal extension (blue arrow: AFX2 main body; green arrow: VELA). (**B**) Three-dimensional reconstruction of contrast-enhanced computed tomography following open graft replacement, showing partial preservation of the VELA suprarenal extension graft.

Follow-up consisted of annual CT imaging and plain abdominal radiography. At the 1-year follow-up, the component alignment was generally well maintained; however, plain radiography revealed a slight reduction of several millimeters in the overlap. The aneurysm diameter showed no change compared with the immediate postoperative imaging. Two years post-procedure, imaging demonstrated complete separation between the AFX2 main body and the VELA extension, resulting in a type IIIa endoleak. Given the marked component separation, endovascular reintervention was deemed infeasible, and open surgical repair was undertaken. Under general anesthesia, a midline laparotomy was performed to access the retroperitoneal space. The VELA extension reached suprarenally, necessitating a clamp across the mid-portion of the VELA itself. Distal control was obtained at both common iliac arteries. Upon opening the aneurysm sac, the complete separation of the components was confirmed (**Fig. 3**). The proximal anastomosis was created by transecting the VELA, removing the metallic stents near the cut edge, and then using the remaining graft fabric as a sewing cuff, to which a 16-mm Dacron graft was sutured. The distal stent graft components were then completely removed. The postoperative course was uneventful, and the patient was discharged on postoperative day 14.

## Discussion

Type IIIa endoleaks originate from the modular junctions of stent grafts.^1)^ In the AFX endograft, these events are particularly associated with inadequate overlap between the AFX2 main body and the VELA extension.^2)^ Hoshina et al. reported overlap reduction in 3.1% of cases within 3 years.^3)^ The manufacturer recommends an overlap exceeding the aneurysm radius plus 20 mm.^4)^ Despite adherence to this recommendation, migration occurred in our case. Oda et al. suggested that shortening at the junction between the fourth stent segment and the bifurcation may induce migration.^5)^ It is conceivable that the overlap length in this case, although initially adequate, progressively shortened over time, falling below the recommended threshold and resulting in sideways displacement.^3)^ In fact, the abdominal plain radiograph at 1 year demonstrated a shortening of several millimeters. In addition, at the 2-year follow-up, shortening at the junction between the fourth stent segment and the bifurcation had become apparent. Given the aneurysm morphology, a longer device should have been selected to secure greater overlap. We judged that a sufficient overlap length existed even on the shorter side of the curved overlap; however, the actual effective overlap may have been shorter than estimated.

Lemmon et al. identified an aneurysm diameter >65 mm as a predictor of overlap reduction.^6)^ Our case met this criterion. Although the mural thrombus was expected to mitigate lateral migration, this effect was not observed. Considering that the thrombus burden can either increase or decrease after EVAR, reliance on thrombus for fixation is unreliable. Furthermore, the proximal neck angulation of 55° was within the Instructions for Use limit (<60°) but near the threshold, possibly contributing to sideways displacement when combined with the aneurysm size. If aneurysm sac shrinkage had been achieved, this type of disconnection might not have occurred. To increase the likelihood of sac shrinkage, embolization of branch vessels such as the lumbar arteries might also have been considered.^7)^

In this case, the change at 1 year postoperatively was minimal, but a sudden complete separation became apparent at 2 years. The timing of type IIIa endoleaks after AFX endograft implantation is variable, ranging from early onset at 1 year to late presentation at 7 years.^8)^ While endovascular reintervention has been reported, open repair remains necessary in cases of complete separation, provided the patient is a suitable surgical candidate. Given the potential for sudden and significant uncoupling, more frequent surveillance should be considered for patients with high-risk anatomical features. In the present case, slight migration was already evident at 1 year postoperatively. Given the absence of aneurysm sac shrinkage, additional evaluation with contrast-enhanced CT and closer outpatient follow-up at shorter intervals should have been considered. To our knowledge, a case of such rapid and extensive separation has not been previously reported, underscoring the importance of vigilant follow-up.

## Conclusion

We describe a case of delayed, complete component separation of an AFX endograft system, leading to a type IIIa endoleak. This case highlights that even with adherence to the manufacturer's recommended overlap, patients with challenging anatomy, specifically a large aneurysm diameter combined with severe neck angulation, remain at high risk for this serious failure, even with a large mural thrombus burden within the aneurysm sac. Therefore, securing a much longer overlap than recommended and implementing a more intensive surveillance protocol should be strongly considered in this high-risk patient subgroup.
